# Role of Environmental Confounding in the Association between *FKBP5* and First-Episode Psychosis

**DOI:** 10.3389/fpsyt.2014.00084

**Published:** 2014-07-17

**Authors:** Olesya Ajnakina, Susana Borges, Marta Di Forti, Yogen Patel, Xiaohui Xu, Priscilla Green, Simona A. Stilo, Anna Kolliakou, Poonam Sood, Tiago Reis Marques, Anthony S. David, Diana Prata, Paola Dazzan, John Powell, Carmine Pariante, Valeria Mondelli, Craig Morgan, Robin M. Murray, Helen L. Fisher, Conrad Iyegbe

**Affiliations:** ^1^Department of Psychosis Studies, Institute of Psychiatry, King’s College London, London, UK; ^2^Department of Health Services and Population Research, Institute of Psychiatry, King’s College London, London, UK; ^3^Department of Neuroscience, Institute of Psychiatry, King’s College London, London, UK; ^4^MRC Social, Genetic and Developmental Psychiatry Centre, Institute of Psychiatry, King’s College London, London, UK; ^5^Department of Psychological Medicine, Institute of Psychiatry, King’s College London, London, UK

**Keywords:** *FKBP5*, psychosis, confounding factors, cannabis, childhood adversity, gene–environment, GWAS, missing heritability

## Abstract

**Background:** Failure to account for the etiological diversity that typically occurs in psychiatric cohorts may increase the potential for confounding as a proportion of genetic variance will be specific to exposures that have varying distributions in cases. This study investigated whether minimizing the potential for such confounding strengthened the evidence for a genetic candidate currently unsupported at the genome-wide level.

**Methods:** Two hundred and ninety-one first-episode psychosis cases from South London, UK and 218 unaffected controls were evaluated for a functional polymorphism at the rs1360780 locus in *FKBP5*. The relationship between *FKBP5* and psychosis was modeled using logistic regression. Cannabis use (Cannabis Experiences Questionnaire) and parental separation (Childhood Experience of Care and Abuse Questionnaire) were included as confounders in the analysis.

**Results:** Association at rs1360780 was not detected until the effects of the two environmental factors had been adjusted for in the model (OR = 2.81, 95% CI 1.23–6.43, *p* = 0.02). A statistical interaction between rs1360780 and parental separation was confirmed by stratified tests (OR = 2.8, *p* = 0.02 vs. OR = 0.89, *p* = 0.80). The genetic main effect was directionally consistent with findings in other (stress-related) clinical phenotypes. Moreover, the variation in effect magnitude was explained by the level of power associated with different cannabis constructs used in the model (*r* = 0.95).

**Conclusion:** Our results suggest that the extent to which genetic variants in *FKBP5* can influence susceptibility to psychosis may depend on other etiological factors. This finding requires further validation in large independent cohorts. Potentially this work could have translational implications; the ability to discriminate between genetic etiologies based on a case-by-case understanding of previous environmental exposures would confer an important clinical advantage that would benefit the delivery of personalizable treatment strategies.

## Introduction

The broad etiological basis of psychosis adds to the expectation that the genetic pathways to the disorder will be similarly diverse. But evidence in support of this theory has been hard to come by in psychosis research. One reason for this is that exposures which may have contributed to the clinical state are rarely documented in genetic research. Gene–environment interaction (GxE) is the archetypal strategy for exploring interplay between genes and environment (1, 2) but is not the only plausible mechanism that may occur. For example, failing to account for diversity of the etiological pathways to psychosis may be contributing to genetic confounding, because a proportion of the genetic variance associated with the psychosis trait will be attached to exposures (such as cannabis), which have a limited distribution in psychosis cohorts (2, 3). Adding to this complexity is the fact that exposure to a risk factor does not impact all members of a cohort equally.

In its present format, genome-wide association (GWA) is biased toward the discovery of associations that are robust to these concerns. In theory at least, one way the range of GWA variants for psychosis could be extended beyond this constraint is by incorporating the environmental structure of the cohort into GWA analyses, as was recently attempted in depression (4). We employ the term, “environmental stratification” to describe the consequences of ignoring this problem, given that it may resemble “population stratification,” a well-recognized phenomenon and source of distortion in genetic studies. Social science has drawn attention to additional higher order complexity in non-genetic risk models for psychosis. For instance, several lines of evidence suggest that interaction between childhood trauma and cannabis smoking should be routinely tested and adjusted for in studies of psychosis (5–7). However, in genetic studies it has not been proven that the etiological composition of a cohort can impede the discovery of underlying associations. One way in which the theory can be tested is by examining the impact of correcting for such structure on the detection of association signals.

With this in mind, we set out to test the credentials of *FKBP5* (*FK506* binding protein 5) as a genetic risk factor for psychosis. *FKBP5* performs a vital physiological function at the gene–environment interface, as it regulates the activity of the hypothalamic–pituitary–adrenal axis (HPA) system. This regulatory function is known to be perturbed by genetic and epigenetic events in some stress-related conditions (8–10). In particular, the link between rs1360780 and psychiatric disorders is endorsed by high-level functional research (9, 10) but it curiously lacks GWA support in any one particular disorder. Two studies have already looked at the possible relevance of *FKBP5* to psychosis. Gawlik et al. (11) tested for an association of rs1360780 with *affective* psychosis, in a study that controlled for group differences in gender, but did not model environmental co-factors. The study failed to provide evidence in support of an association with psychosis. The second, more recent study by Collip et al. (12) found limited evidence of an interaction between *FKBP5* and childhood trauma. One explanation for this could be that the variable level of control for environmental effects exercised in the study limited the ability to detect the interaction. Moreover, this study focused on symptoms and did not explore full-blown psychotic disorder as an outcome.

We therefore set out to test for a main effect of rs1360780 on the presence of psychotic disorder. Our approach incorporates an attempt to reduce environmental confounding and assess the consequence this has on the genetic association. Specifically, we adjust for cannabis use and childhood adversity, with the type of adversity informed by the statistical power in the available sample. Both factors are known to be associated with psychosis in the original population from which the sample is drawn (3, 13, 14).

## Materials and Methods

### Recruitment

**Figure 1:**
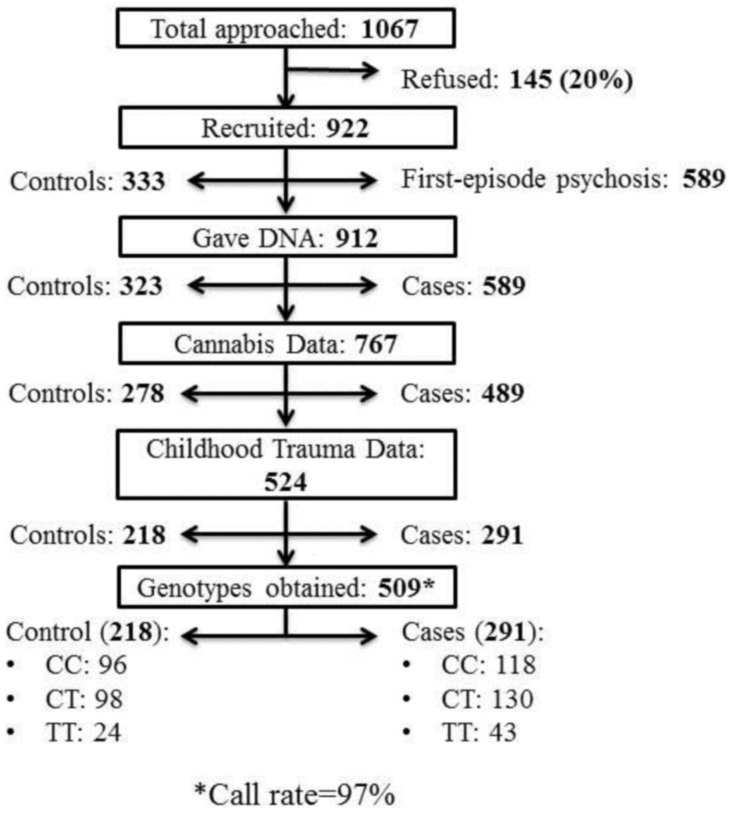
**Flow diagram and numerical breakdown of outcomes for subject recruitment and genotyping**.

Patients (*n* = 291) aged 18–65 years and presenting with their first-episode of psychosis to psychiatric inpatient wards in Lambeth, Southwark, and Croydon (South London, UK), between December 2005 and April 2011 were invited to take part in the study. All patients that met ICD-10 (15) criteria for non-organic psychosis (codes F20–F29 and F30–F33) were considered eligible. Controls (*n* = 218) were drawn from the same geographical areas as cases. All controls were asked to complete a Psychosis Screening Questionnaire (PSQ) (16) in order to determine the possibility of having an undiagnosed psychotic disorder. Controls who responded positively to items on the PSQ were excluded if they were subsequently found to meet diagnostic criteria for a psychotic disorder or reported a history of psychosis. Potential cases and controls with severe learning disability (IQ < 50) or poor English fluency were excluded. An overview of recruitment and attrition can be found in Figure 1.

### Ethics

The study cohort used is drawn from the Genetics And Psychosis (GAP) study, which was granted ethical approval by the South London and Maudsley and Institute of Psychiatry Local Research Ethics Committee (Ethics reference number: 05/Q0706/158). All cases and controls gave informed written consent after reading a detailed information sheet.

### Cannabis Experiences Questionnaire

A simple yes/no question about life-time cannabis use was included in the original GAP questionnaire in 2005. A more detailed history of illicit drug use for each subject was introduced when the modified version of the Cannabis Experiences Questionnaire was implemented in 2006 (17). Three primary measures of cannabis use were used in the study: life-time cannabis use (never vs. at least once), frequency of use (life-time, yearly, monthly, weekly, or daily use), and type of cannabis used (hash-like or skunk-like). The most important discrepancy between hash-like and skunk-like forms of cannabis lies in the content of the psychoactive agent as Tetrahydrocannabinol (THC) content varies from 4% in hash to 18% in skunk (3). The later introduction of the detailed cannabis scale to the GAP study has repercussions for sample size, which become apparent in analyzes that use detailed cannabis measures. For example, the maximum available *n* for the “frequency of use” measure was 234 in the subset of GAP with available data on adversity in childhood, whereas for the “life-time use” measure data on 455 was available in the same subset.

### Childhood Experience of Care and Abuse Questionnaire

Childhood adversity was assessed using the Childhood Experience of Care and Abuse Questionnaire (CECA.Q) (18). This is a self-report retrospective measure designed to capture traumatic experiences occurring before the age of 17. Summary variables were created for parental separation, physical, and sexual abuse [see Fisher et al. (13) for full details]. The scales used to measure each variable were dichotomized using the most conservative published cut-off points (18). This ensured that scores in the affirmative reflected a reasonable level of severity in the analysis and is consistent with previous analyses conducted with this measure (13, 18). The CECA.Q has previously been shown to be reliable in patients with psychosis (19).

### Validation of self-reported ethnicity

Genetic ancestry was derived from a panel of 57 ancestry informative genetic markers. These were genotyped using iPLEX technology developed for the MassArray platform (Sequenom Inc., San Diego, CA, USA). Further information on the makeup of the marker panel is available on request. Ancestry scores were derived using the program Structure (20) to implement a model-based (Markov Chain Monte Carlo) clustering algorithm. Further details of the methodology used are described in a previous paper (21).

### Genotyping

DNA material from buccal swabs and blood samples was obtained from a total of 509 subjects (291 cases and 218 controls). DNA was extracted using a standard phenol–chloroform extraction procedure. A Taqman SNP assay (Applied Biosystems, USA) was used to report the presence of either one or both alleles (C and T) at the rs1360780 locus. Genotypic concordance was checked for a subset of cases for which cheek swab and blood DNA was available (*n* = 126). Concordance between the two sources was 100%.

### Statistical analysis

Power calculations were performed in Quanto, version 1.2.4 (University of Southern California, USA). All other analyses were conducted using STATA, release 12 (STATACorp LP, USA). Genotypic coding for the genetic analysis reflected either additivity (CC vs. CT vs. TT) or dominance (CC vs. CT + TT). Chi-square tests were used to identify variables confounded with either diagnostic status or genotype. Logistic regression analyses incorporated these variables into a model that included age, gender, and genetic ancestry. A single measure of childhood trauma was selected for use in these analyses on the basis of statistical power, whereas three cannabis measures (life-time use, cannabis type, and frequency of smoking) were used in the analysis for comparison purposes. In this study G × E interaction is defined as a departure from multiplicativity, unless otherwise stated. Consideration of E × E effects was prompted by three previous studies in this area (5–7). These studies all report interactions between cannabis smoking and childhood adversity that transcend differences between additive and multiplicative scaling. The E × E model used considered effects on a multiplicative interaction scale.

## Results

A total of 509 out of 524 possible genotypes were obtained. This corresponds to an overall call rate of 97%. Allele frequencies (for the rs1370780-*T* allele) were compared with HAPMAP reference populations (22). Allele frequencies deviated only slightly from the expected proportions in Caucasians and Africans. Neither deviation was statistically significant (*X^2^* ≤ 1.149; *p* ≥ 0.41). Genotypes were in the expected proportions, given allelic frequency (*X^2^* = 0.12; *p* = 0.74). Therefore, no evidence of bias in the derivation of genotypic data could be detected.

The proportion of male psychosis cases was significantly greater compared to controls (60.1 vs. 50.5%, *X^2^* = 4.3; *p* = 0.04), but the median age at assessment did not differ between the two groups (27 vs. 26 years, Z = 0.006; *p* = 0.95). The comparison groups used in this study were diverse in terms of the self-reported ethnic labels used (Fisher’s Exact *X^2^* = 24.25; *p* = 0.001). For this reason, genetic ancestry scores are used henceforth to index quantitative ancestry. There was a high degree of correspondence between self-reported ethnicity and mean ancestry scores (Table S1 in Supplementary Materials). The main effects of genetic and non-genetic risk factors on psychotic disorder are presented in Table 1.

**Table 1:** **Main effects of environmental risk factors and *FKBP5* on psychotic disorder**.

Environmental variable	Variant	*N*	Unaffected controls	Psychosis cases	Association with psychotic disorder	
			*n* (%)	*n* (%)	Unadjusted OR (95% CI); *p* value	Adjusted* OR (95% CI); *p* value
Separation from parent before 17 (*n* = 493)	No	261	138 (63.6)	123 (44.6)	2.17 (1.5–3.1); <0.0001	1.96 (1.3–2.9); 0.001
	Yes	232	79 (36.4)	153 (55.4)	
Physical abuse before 17 (*n* = 496)	No	399	183 (84.3)	215 (77.3)	1.57 (0.99–2.49); 0.06	1.30 (0.8–2.12); 0.29
	Yes	97	34 (15.7)	63 (22.7)	
Sexual abuse before age 17 (*n* = 496)	No	429	192 (88.5)	237 (85.0)	1.36 (0.80–2.31); 0.26	1.39 (0.79–2.46); 0.25
	Yes	67	25 (11.5)	42 (15.0)	
Life-time cannabis use (*n* = 455)	No	236	105 (56.5)	131 (48.7)	1.37 (0.94–1.99); 0.10	1.31 (0.87–1.96); 0.19
	Yes	219	81 (43.5)	138 (51.3)	
Frequency of cannabis use (*n* = 234)	Non-daily use	149	95 (82.0)	54 (45.8)	5.36 (2.95–9.72); <0.0001	5.86 (3.02–11.37); <0.0001
	Daily Use	85	21 (18.0)	64 (54.2)	
Type of cannabis use (*n* = 228)	Hash	84	59 (53.2)	25 (21.4)	4.16 (2.34–7.44); <0.0001	3.44 (1.87–6.32); <0.0001
	Skunk	144	52 (46.9)	92 (78.6)	
*FKBP5* rs1360780 (*n* = 509)	CC	214	96 (44.0)	118 (40.5)	1.19 (0.92–1.55); 0.18	1.10 (0.83–1.46); 0.5
	CT	228	98 (45.0)	130 (44.7)	
	TT	67	24 (11.0)	43 (14.8)	

*Adjusted for sex, age, and genetic ancestry. CI, confidence interval; OR, odds ratio.

Of the three measures of adversity shown in Table 1, only parental separation was significantly associated with psychosis after adjustment for sex, age, and genetic ancestry (OR_adj_ = 1.96, *p* = 0.001).

Although a higher proportion of cases admitted to having used cannabis during their life-time (Table 1), this trend did not reach significance at the 5% level (OR_adj_ = 1.31, *p* = 0.19). However, daily cannabis use was significantly higher among cases than controls (54.2 vs. 18.1%) compared to all other intake regimes combined (OR_adj_ = 5.86, *p* < 0.0001). There was also increased use of THC-enriched “*skunk-like*” varieties of cannabis among cases (78.6 vs. 47%; OR_adj_ = 3.44, *p* < 0.0001). No main effect between *FKBP5* and psychotic disorder was observed in either crude or adjusted models.

Overall, the profile of cannabis usage in this cohort concurs with that of a larger, partially overlapping dataset (3). We found no deviation in usage profile (frequency, type) from the larger dataset from which this cohort was derived (frequency of use: *X^2^* = 1.6, *p* = 0.8; cannabis type: *X^2^* = 2.8, *p* = 0.1). In summary, the associations between parental separation, cannabis use, and psychotic disorder in this sample are consistent with our previously published data (3, 14), on socio-environmental risk factors for psychosis, thus, there is no evidence to suggest that sampling bias has altered the characteristics of the sample used in the analyses that follow.

### Association between *FKBP5* and environmental factors

Table 2 shows the relationship between genotype at locus rs1360780 and environmental risk factors in this sample. Out of the three types of adversity and three cannabis measures initially included, only parental separation shows evidence of association with genotype, although the effect only borders statistical significance (*X^2^* = 6.13, *p* = 0.05). Further group-specific analyses suggest that the association is exclusive to cases (*X^2^* = 6.9, *p* = 0.03) and not controls (*X^2^* = 1.06, *p* = 0.6); this implies G × E between *FKBP5* and parental separation, as opposed to gene–environment correlation.

**Table 2:** **Association between environmental risk factors and *FKBP5* genotype**.

Environmental variable	Variant	*N*	CC	CT	TT	Genotypic association*
			*n* (%)	*n* (%)	*n* (%)	*X^2^* (*p* value)
Physical abuse before age 17	No	408	168 (81. 6)	179 (80. 0)	52 (78. 9)	0. 44 (0. 80)
	Yes	100	38 (18. 4)	45 (20. 0)	14 (20. 1)	
Sexual abuse before age 17	No	203	79 (38. 54)	106 (47. 5)	18 (28. 1)	2. 19 (0. 34)
	Yes	289	126 (61. 5)	117 (52. 5)	46 (71. 9)	
Life-time cannabis use	No	236	95 (50. 53)	110 (53. 92)	31 (49. 21)	0. 66 (0. 72)
	Yes	219	93 (49. 47)	94 (46. 08)	32 (50. 79)	
Frequency of cannabis use	Low	149	61 (62. 2)	69 (68. 3)	19 (54. 3)	2. 85 (0. 24)
	High	85	37 (37. 8)	32 (31. 7)	16 (45. 7)	
Type of cannabis use	Hash-like	84	36 (35. 3)	37 (37. 4)	11 (40. 7)	0. 29 (0. 86)
	Skunk-like	144	66 (64. 7)	62 (62. 6)	16 (59. 3)	
Separation from either parent before 17	No	261	106 (51. 5)	129 (57. 8)	26 (40. 6)	6. 13 (0. 05)
	Yes	232	100 (48. 5)	94 (42. 2)	38 (59. 4)	
Separation from parent before 17 (CONTROLS)	No	138	62 (65. 2)	63 (64. 29)	13 (54. 17)	1. 06 (0. 59)
	Yes	79	33 (34. 74)	35 (35. 71)	11 (45. 83)	
Separation from parent before 17 (CASES)	No	123	44 (41. 53)	66 (51. 94)	13 (31. 71)	6. 9 (0. 03)
	Yes	153	67 (58. 47)	59 (48. 06)	27 (68. 29)	

*Calculated under an assumed additive genotypic model using a 3 × 2 (2df) chi-square test.

### Interaction between cannabis use and childhood adversity

The need to account for interaction between life-time cannabis use and childhood adversity in this sample (5–7) was assessed objectively by attempting to replicate the interaction using chi-square tests. However, we failed to find statistical support for the interaction in the current dataset (Table S2 in Supplementary Materials; *p* ≥ 0.49; *n* ≥ 420). Based on this result interactions between environmental variables (E × E) were excluded from consideration in the full logistic model applied in the next section.

### Effect of adjusting for environmental exposure on *FKBP5* association with psychotic disorders

The full logistic model we applied included terms for parental separation, cannabis use, sex, age, genetic ancestry, and the interaction between *FKBP5* and parental separation (see Table 2). The outcomes of interest were the logistic regression statistics, which assess the association between *FKBP5* and psychotic disorder. Results are presented in Table 3.

**Table 3:** **Effect of *FKBP5* on psychosis caseness after adjusting for environmental exposures**.

Genetic model	Model no.	Basic model*, plus	*n*	Rs1360780	
				Main effect; OR (95% CI);	Interaction effect; OR (95% CI);
				*p* value	*p* value
Additive	1	Life-time cannabis use	420	1.29 (0.89–1.87); 0.18	0.57 (0.26–1.25); 0.16
	2	Frequency of cannabis use	227	1.63 (0.96–2.77); 0.07	0.47 (0.16–1.41); 0.18
	3	Type of cannabis used	220	1.54 (0.87–2.7); 0.14	0.39 (0.13–1.20); 0.10
Dominant	4	Life-time cannabis use	420	1.54 (0.90–2.93); 0.11	0.51 (0.22–1.17); 0.11
	5	Frequency of cannabis use	227	2.81 (1.23–6.43); 0.02	0.31 (0.09–1.04); 0.06
	6	Type of cannabis used	220	2.02 (0.89–4.65); 0.10	0.34 (0.08–1.04); 0.07

*Models 1–6 vary in terms of (i) which cannabis variables are modeled and (ii) the genetic effect model applied (i.e., additivity vs. dominance)*.

*Basic model: parental separation, genetic ancestry, sex, age, the main effect of rs1360780, and the SNP × separation G × E term.

*(CI, confidence interval; OR, odds ratio)*.

A trend effect found under the additive genetic model (Table 3, model 2) was driven by the heterozygote (*CT*) genotype class. This was enough to prompt a recode of genotype data in order to allow re-estimation of the same parameters under the dominance genetic model (models 4–6). This had the added advantage of addressing the statistical constraint imposed by the low representation of the *TT* genotype class (*n* = 67) (see Table 1). This recoding brought main effects and interactions into sharper focus, especially in model 5 (Table 3) where the main effect of *FKBP5* was strongest (OR = 2.81, *p* = 0.02). The direction of this effect accords with previous studies in which the *T* allele mediates increased risk (see Table S3 in Supplementary Materials). The interaction effect peaks in model 5, and is directionally consistent across models 1–6. The *T* allele confers protection from risk in this interaction. Overall, the inclusion of both main and interaction terms in the model significantly benefits model-fit performance (Likelihood Ratio Statistic = 6.12, *p_two-tailed_* = 0.047, *df*  = 2).

The interaction was reinterpreted using the additive scale (23). The interaction effect on psychosis caseness again proved to be significant at the *p* < 0.05 level (data not shown). This suggests that the significant interaction terms in Table 3 are not merely the consequence of logarithmic scaling, as has previously been proposed (24). The interaction result is therefore worthy of independent replication efforts.

### Stratified tests

Table 3 suggests that a main effect of *FKBP5* is obscured by etiological heterogeneity within the sample. We sought further clarification of the result from a stratified analysis. The importance of parental separation to the genetic model can be confirmed by the data shown in Table 4, which demonstrates the effect of the locus on presence of psychotic disorder and its dependence on exposure status.

**Table 4:** **Stratification of association between *FKBP5* and psychotic disorder by parental separation**.

Parental separation	Adjusted effect of rs1360780; OR (95% CI), *p* value
Yes (*n* = 114)	0.89 (0.37–2.17), 0.80
No (*n* = 114)	2.8 (1.21–6.60), 0.02

Genetic terms used in the analysis are dominance-coded and *adjusted for sex, age, genetic ancestry, and frequency of cannabis use. The partitioning of parental separation in this stratified analysis makes the G × E term used in the logistic model (FKBP5 × parental separation) redundant in this analysis. CI, confidence interval; OR, odds ratio.

### Genetic power of the cannabis variables

**Figure 2:**
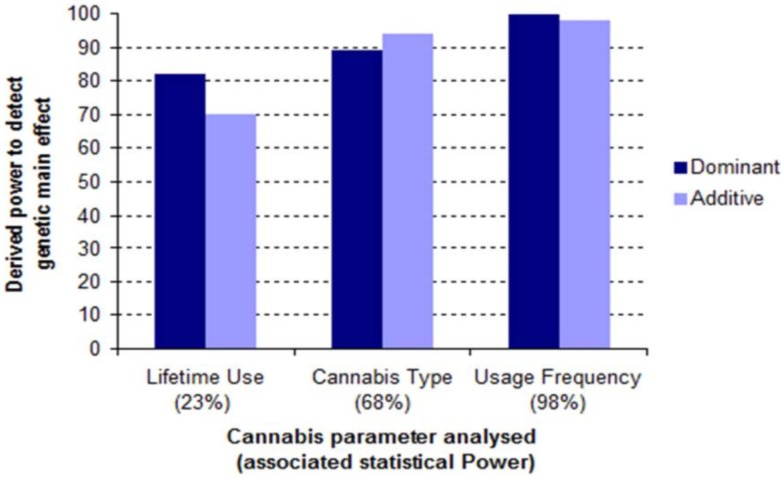
**Environmental vs. genetic power in the conditioned analyses of association with psychotic disorder**. Life-time cannabis use, cannabis type, and frequency of cannabis use are associated with low (23%), intermediate (68%), and high (98%) statistical power (respectively) (Table S5 in Supplementary Materials). These power analyses confirm that conditioning on the more discriminating measures of cannabis use leads to larger genetic effects and, correspondingly, better detection efficiency (genetic power). The same pattern was also seen when these analyses were repeated under an additive genetic model.

Table S4 in Supplementary Materials presents the power of the three cannabis constructs used in these analyses. Genetic power to detect the genetic main effects shown in Table 3 does not so much depend on sample size (*r* = −0.78) as it does on the *power* of the cannabis construct used (*r* = 0.95) (Table S5 in Supplementary Materials). This is also illustrated in Figure 2. Hence, genetic power is maximized when “frequency of cannabis use” is included in the covariate model and is weakest when the measure is substituted with “life-time cannabis use.” This dosage effect of non-genetic power on genetic outcomes seems to be consistent with our hypothesis that the environmental effects may need to be attenuated in order to demonstrate associations between rs1360780 and psychosis.

## Discussion

This study attempted to investigate whether the etiological heterogeneity typically found in large clinical cohorts is a root cause of the uncertainty regarding *FKBP5*’s relevance to psychosis. This uncertainty includes a lack of GWA support and the mixed performance of the target locus in one recent study (12). Our approach to overcoming the heterogeneity “problem” involved an analytical process in which terms for environmental exposure were incorporated into a logistic regression model. An association between rs1360780 and psychosis was not detected until cannabis use and parental separation had been adjusted for in the analysis (OR = 2.81, *p* = 0.02). The genetic main effect found is directionally consistent with that of the same locus in other (stress-related) clinical phenotypes (8–10). Moreover, we have found that the magnitude of the effect varies with the power of the different cannabis-related measures included in the model (*r* = 0.95). The statistical interaction between rs1360780 and parental separation was confirmed in a stratified analysis (OR = 2.8, *p* = 0.02 vs. OR = 0.89, *p* = 0.80).

One interpretation of the findings described is that they represent evidence of a genetic delineation between different etiological pathways to psychosis. This would suggest a need to acknowledge and understand the role of the environment in shaping and resolving genetic liability to mental illness including psychosis (25). Advances such as the polygenic risk score, make this prospect more feasible than ever (26, 27). The example of *FKBP5* potentially demonstrates that greater variety and subtlety of signal detection could be achieved (and hence, the interests of translational medicine best served) if our awareness of non-genetic risk factors is harnessed in the discovery process. Failure to accomplish this may come at the expense of the translational goals of psychiatric research (28, 29) as “missing heritability” is a complex void which genome only approaches are unlikely to explain in full, irrespective of ever improving sample sizes (30). This is because the etiological complexity we have outlined (and its potentially negative impact on signal detection) does not diminish with scale.

There are important contrasts to consider between our results and those of a previous study of psychosis focused on the same SNP (12). Firstly, the studies differ with respect to the types of effect sought: the effect of interest in Collip et al. (12) was a G × E *interaction* involving the rs1360780 locus and a much broader trauma definition than was used here, while the focus of the present study was primarily genetic *main effects*. Moreover, they focused specifically on psychotic symptoms whereas our outcome was presence of a clinically relevant psychotic disorder. However, we did find evidence of an interaction between *FKBP5* and parental separation. Therefore, the two studies are consistent in this regard, although specific effects on risk appear to be discrepant between the studies.

Despite the different hypothesis explored by each study (i.e., main effects vs. interaction), the theory of environmental confounding could explain why interactions mediated in the vicinity of rs1360780 showed poor consistency in the analyses of Collip et al (12). In the dominance models of Table 3, interaction effects are as dependent on the power of cannabis measures as main effects (Table S4 in Supplementary Materials and Figure 2). Thus, the interaction is under strong potentiation when “frequency of cannabis use” is included with “parental separation” in the confounder model (Table 3: OR = 0.31, *p* = 0.06). This trend also seems to be reflected in the results of Collip et al. (12): in their first two analyses, which control for stressful life events (models 1 and 2), interaction effects range from suggestive to significant (*p* < 0.08). In models 3, 4, and 5, statistical evidence of interaction is generally weaker (*p* > 0.1), this is seemingly because adjustment for the confounding effect of stressful life events was not implemented in this set of analyses. In total, Collip et al. (12) conducted five separate analyses, spanning sub-clinical and biological expressions of psychosis-related traits. Two variants in close genetic proximity to rs1360780 (*r* ≥ 0.80) were found to be the most consistent across all five models. Our own analytical design additionally factors-in cannabis-related characteristics of the cohort while cannabis does not feature in the models of Collip et al. (12). However, as an important risk factor for psychosis (3) that also influences the sub-clinical expressions of this trait (31, 32), we suggest it is appropriate to adjust for its potentially confounding effects.

This study has a number of methodological limitations that should be taken into account when interpreting these findings. We were unable to control for symptoms of depression in our cohort, because such data was not collected. Previously reported main and interaction effects of rs1360780 span the depression phenotype (9, 33, 34), therefore, a failure to control for this as a further possible confounder is a limitation of this study. Another limitation is the likelihood that effects in this sample (and hence, our power to detect them) are overestimated, due to the relatively small sample used. Hence, larger cohorts are required to validate these findings. Additionally, the *p* values reported in this study have not been corrected for multiple-testing. The reasons for this are twofold: (i) a balanced penalization strategy should take into account the compelling evidence that made rs1360780 our preferred genetic candidate (e.g., 8–10, 33, 34); and (ii) the bulk of the additional testing relates to the formulation of our environmental model. For this we applied a principled approach, which sought to identify and use the most statistically relevant adverse childhood exposure in the cohort. We then explored the relationship between power and genetic outcome across all three cannabis measures. Finally, the study has been performed on a restricted (hospitalized) patient population within an inner-city area and controls were not randomly selected, though they were broadly matched across several demographic criteria (age, education, and ethnicity). This may limit the generalizability of the findings. Future work could include further evaluation of our findings in more representative psychosis samples and community-based populations.

## Conclusion

Although, we were able to detect the hypothesized effect of *FKBP5* on presence of psychotic disorder, the fact that our ability to detect this signal depended on controlling for environmental exposures suggests that *FKBP5’s* contribution may vary depending on the etiology relevant to each case. Translation of these findings into clinical practice might one day serve the need to factor individual profiles of exposure into calculations of personal risk. The ability to accurately pinpoint or discriminate the relevant genetic etiology based on a case-by-case understanding of environmental pathology, could provide an important clinical advantage by strengthening personalized medicine approaches within psychiatry.

## Conflict of Interest Statement

The authors declare that the research was conducted in the absence of any commercial or financial relationships that could be construed as a potential conflict of interest.

## Supplementary Material

The Supplementary Material for this article can be found online at http://www.frontiersin.org/Journal/10.3389/fpsyt.2014.00084/abstract
