# Supplementary material for: Role of Environmental Confounding in the Association between FKBP5 and First-Episode Psychosis
Source: Front Psychiatry. 2014 Jul 17;5:84. doi: 10.3389/fpsyt.2014.00084 (PMC4101879; doi:10.3389/fpsyt.2014.00084)
Supplement: Supplementary file 1 [file Presentation1.PDF]

Supplementary Material

Table S1. Self-reported versus Genetic Ancestry in psychosis cases and unaffected controls

Self-reported ethnic group	*n*	Ancestry score for corresponding genetic population*
White Caucasian	158	0.85
Asian	27	0.88
Black African	83	0.99

**Based on a three-way ancestral axis constructed to be informative for Black African, European Caucasian and Asian ancestry.*

Table S2. Lifetime cannabis use x childhood adversity interactions for psychotic disorder

Type of adversity	Exposure	Lifetime cannabis use: Psychosis cases (n=291) Yes n (%)	Psychosis cases (n=291) No n (%)	Association	Controls (n=218) Yes n (%)	Controls (n=218) No n (%)	Association	ExE effect* OR [95% CI]
**Separation from parent before 17 (n=451)**	Yes	83 (56.5)	54 (45.5)	χ^2 = 3.23 *p* = 0.07	30 (49.2)	50 (40.3)	χ^2 = 1.3 *p* = 0.25	0.80 [0.35-1.82] *p* = 0.50
	No	64 (43.5)	65 (54.6)		31 (50. 8)	74 (59.7)		
**Physical abuse before 17 (n=454)**	Yes	39 (61.9)	99 (48.1)	χ^2 = 3.7 *p* = 0.05	19 (61.3)	62 (40.3)	χ^2 = 4.64 *p* = 0.03	0.59 [0.21-1.62] *p* = 0.31
	No	24 (38.1)	107 (51.9)		12 (38.7)	92 (59.7)		
**Sexual abuse before age 17 (n=454)**	Yes	23 (54.8)	115 (50.7)	χ^2 = 0.24 *p* = 0.6	15 (65.2)	66 (40.7)	χ^2 = 4.9 *p* = 0.03	0.46 [0.14-1.46] *p* = 0.19
	No	19 (45.2)	112 (49.3)		8 (34.8)	96 (59.3)		

**Adjusted for age at assessment, gender, genetic ancestry, and the corresponding main effect terms. CI, confidence interval; OR, odds ratio.*

Table S3. Overview of studies of rs1360780 in psychiatry

Author	Year	Effect Type	Population	Disorder	Sample size	Outcome	PubMed ID
Sarginson et al	2010	Main Effect	USA	Depression	246	Non-Significant	19676097
Binder et al	2008	GxE	US (African-American)	PTSD	900	Significant	18349090
Roy et al	2010	GxE	US (African-American)	Suicidality	706	Significant	20090668
Brent et al	2010	Main Effect	USA	Suicidality	176	Significant	20008943
Gawlik et al	2006	Main Effect	Caucasian	Affective Psychosis	248	Non-Significant	17081296
Zobel et al	2010	Main Effect	German	Unipolar depression	284	Significant	20047716
Kirchheiner et al	2008	Main Effect	German	Depression	179	Significant	18597649
Ising et al	2008	Main Effect	German	Mood and Anxiety disorders	64	Significant	18702710
Appel et al	2011	GxE	German	Depression	2157	Significant	16311898
Lekman et al	2010	Main Effect	European and African-American	PTSD	2262	Significant	18803430
Bevilacqua et al	2012	Main Effect	Italian	Aggressive behaviour	583	Significant	22213790
Supriyanto et al	2010	Main Effect	Japanese	Suicide	447	Significant	21112363
Koenen et al	2005	Main Effect	USA (Black and White)	Peri-traumatic dissociation	56	Significant	16088328
Binder et al	2004	Main Effect	German	Depression	294	Significant	15565110
Lavebratt et al	2010	Main Effect	Swedish	Depression	8613	Significant	20226536
Zimmermann et al	2011	GxE	German	Major Depression	884	Significant	21865530

Table S4. Comparison of detection power for cannabis-related variables

	CECA.Q sample		Full sample	
**Cannabis risk Model**	**Critical Values (adjusted*) OR [95% CI]**	**Estimated power**	**Critical Values (adjusted*) OR [95% CI]**	**Estimated power**
Frequency of use	n=234<br>5.86 [3.02-11.37]<br>*p* = 0.0001	98%	n=314<br>4.34 [2.58-7.29]<br>*p* <0.0001	97%
Lifetime use	n=455<br>1.3 [0.87-1.96]<br>*p* = 0.19	23% at P=0.05	n=666<br>1.03 [0.73-1.45]<br>*p* = 0.87	7% at P=0.05
Type of cannabis used	n=228<br>3.44 [1.87-6.32]<br>*p* = 0.0001	68%	n=275<br>5.00 [2.86-8.76]<br>*p* <0.0001	99%

*Results in this table relate to the conditioned environmental analyses presented in Table 1 of the main paper and additional analyses performed in the wider GAP sample with available cannabis data (n=666). *Adjusted for age at assessment, gender and genetic ancestry. CECA.Q, Childhood Experience of Care and Abuse Questionnaire; CI, confidence interval; OR, odds ratio.*

Table S5. **Statistical power for detecting genetic effects for different cannabis variables**

**Genetic model**	**Power of derived genetic effect**		
	**Lifetime use**	**Cannabis type**	**Frequency of use**
**Dominant**	82%	89%	99%
**Additive**	70%	94%	98%

The alpha used to generate these estimates (α=0.0001) is based on the adjusted estimates provided in Table 1 of the main paper.
